# The Value of Statistical Information to the Dentist

**Published:** 1862-03

**Authors:** Jno. C. K. Crooks


The value of Statistical Information to the Dentist. —*By Jno. C. K. Crooks, M. D.*—Has it never occurred to the scientific dentist, who is laboring for the best results in his operations, of how much value an abundant statistical knowledge would be to him, as an elucidation of the subjects he may be pursuing? Cases from his own storehouse may be ever so perfectly at his command, yet his good judgment at once warns him of the utter fallaciousness of limited statistics. *Accuracy*, he knows, can only be obtained by the collection of a large number of facts bearing upon the point in question. In matters so various and complicated as those connected with vitality, in relation to which "nothing is little," all the observations of all the profession are required. Take, for instance, that much mooted question, "fang filling," "filling of the nerve cavity," and of how much consequence would be the history of a thousand cases of each method of operating, with the percentage of successes and failures expressed mathematically! And if one thousand such cases would be of so much value, how much more would be the importance of ten or twenty thousand—the *certainty* of the deductions becoming more and more trust-worthy, till, so far as any *practical* value is concerned, the subject might be forever put to rest, or until some new discovery should open the investigation afresh, when old facts would be as available as ever! If, then, when *doubt* obscures the way, (and we long for something more reliable than our present resources furnish us,) statistical information looks so like a deliverance, why can we not, like rational men, begin a system of registration, which, though it may not *immediately* bring the looked-for good, may one day avail us something, or at least produce a record that shall enlighten those who may come after, and teach them that labor of patience which always brings about great results?

Were there ten thousand dentists in our country, and one in ten only were willing to enter into the spirit of a *system* of *registration*, we should have one thousand every day doing something of more or less value to the profession. If they saw but four cases a day, four thousand *facts* would be daily placed in a tangible shape ready for future use. Every night each one of these registrars could have the satisfactory reflection that he had availed himself of the occurrences of the day to contribute his share of materials, which, by careful generalization, might result in the formation of principles approxi mating to *certainty.*

Considering the rapid strides dentistry has made in the last twenty years, is it not now time for theorising and speculation to cease? We have to do with first principles—we have been "fed milk" long enough; let us now make a trial of "strong meat," and discover if the profession and the public are not prepared for it. We can improve the science of dentistry, operative dentistry more particularly, only upon the inductive method and the collection of the greatest number of facts. To our State and National Associations must we look for the inauguration of this great measure—*great,* because of incalculable value to science and of vast importance in producing a uniformity of practice and a uniformity of results. Had we more uniformity in dental practice, a *standard* in operations, the *people themselves* would become informed upon the subject, and the quack would no longer find material so abundant upon which to palm off his jugglery. Statistics would give us this, and when a principle was once *established,* he who would dare to act otherwise than in harmony with such principle would be like the surgeon who, with all the facts before him, with past experience as his guide, would begin the treatment of an aneurism by thrusting a knife into the sac! To this condition of things, medicine owes most of her dignity. Take, as an illustration, the collection of facts bearing upon the different systems of practice, from time to time, in cases of *placenta prœvia.* How the mortality to mother and child were again and again reduced by a more enlightened method of procedure, till facts elicited from thousands of cases have reduced uncertainty to certainty, so that he who *will not* inform himself concerning the present state of this branch of obstetrical science tampers with human life, and may subject himself to the liability of a *criminal* prosecution.

What medicine *has* done, dentistry *can do!* Almost absolute certainty *can* be attained, and there need thus be no more groping in darkness. Give us the *facts,* and the deductions will immediately follow. For the facts, we must rely upon the individual members of an intelligent, enterprising and scientific profession. We should then no longer be drones in the hive of professional usefulness, lazily sucking up the sweets deposited by the patient "workers" in our ranks.

Here is a field ample in dimensions for all, and, unless *all labor*, there will necessarily be furnished but little or no harvest.

But statistical information in dentistry should not be confined alone to subjects directly connected with our *practice.* The *etiology* of the many diseases, to treat which it is the province of the dentist, yet dwells greatly in obscurity. Can nothing be done in this direction also by statistics? Can not *conservative* dentistry here find a valuable support? Has medicine done nothing in the same department of inquiry? If she can work with success, certainly *dentistry* ought, for the ground is much more limited in extent, and just as susceptible of cultivation! These being facts well known to the profession, why speak longer of the value of statistical information to the dentist?

The importance of dental statistics being established, what plan shall be pursued in order to obtain the *material* for generalization? At present, we know of no better than that adopted by the New York State Medical Society, viz.: to furnish blanks, sufficient to make a monthly record of the cases occurring in one year's practice, to every member; these blanks to be carefully filled out with a history of the cases presented in the practice of each person so furnished, and returned at the close of the year to the Secretary of the Society, to be by him, aided by competent assistants, placed into such tabular form as, by a glance, the desired information may be obtained.

Having often, in our own practice, seen the necessity of some more reliable data than we at present possess, it has caused us to give utterance to the above. We trust some one, more competent than ourselves, will pursue the subject further.—*Dental Cosmos.*

